# Knowing You, Knowing Me (KYKM): an interactive game to address positive mother-daughter communication and relationships

**DOI:** 10.3389/fpsyg.2014.00721

**Published:** 2014-07-11

**Authors:** Mary Katsikitis, Christian Jones, Melody Muscat, Kate Crawford

**Affiliations:** ^1^Psychology Department, School of Social Sciences, University of the Sunshine CoastSippy Downs, QLD, Australia; ^2^School of Public Health, Tropical Medicine and Rehabilitation, James Cook UniversityCairns, QLD, Australia; ^3^Eviva Pty Ltd.Maleny, QLD, Australia

**Keywords:** gaming, communication, relationships, positive psychology

## Abstract

This technical report describes an interactive game environment designed to bring mothers and their adolescent daughters together to discuss three issues that have previously been shown in the literature to be of concern to families, as young girls transition from middle childhood to the adolescent years. The game is called Knowing you, Knowing me or KYKM, and is used to help mothers and daughters discuss the following three topics: positive communication skills, relationship building, and managing risky behaviors in the social environment. As the game remains untested, its limitations and future implications of its utility are discussed.

## Introduction

The aim of this project is to provide a positive communication game via the medium of web-based cartoon characters and “sms” messaging to inform mothers and daughters about positive communication, relationship building, and minimization of risky behaviors. The *Knowing you, Knowing me, or the KYKM* project has been developed as a multimedia gaming and mobile resource to increase the positive communication skills of adolescent females and their mothers during this time of developmental transition.

Much of the focus of mother-daughter relationships in the literature has been on strategies to manage and deal with negative adolescent behavior such as identity and attachment issues (Benson et al., 1992), conflict (Steinberg, 1987), violence (Caprara et al., 2002), parental satisfaction (Erel and Burman, 1995), divorce and remarriage (Orbuch et al., 2000), and antisocial behavior (Loeber et al., 1998). This deficit-based approach has been replaced in recent times by the strengths-based approach of positive psychology (Katsikitis et al., 2013a) and is the focus of this paper.

There is a substantial body of evidence endorsing the mother-daughter relationship and the influence that a positive, stable bond has on females throughout their lives (Boyd, 1989; Herman, 1989). The research evidence also suggests that it is the quality of the relationship and interactions between mother and daughter and positive parenting practices that contribute to the psychosocial adjustment of the adolescent girl (Barber et al., 2001). The KYKM project addresses these issues using an innovative, game-based design, which brings mothers and daughters together, builds communication and relationship skills, and uses positive parenting practices to mitigate risk taking behaviors in this young population.

Successful intervention programs meet the needs of parents as well as their children with the best impact being for mothers and daughters in programs that facilitate interaction between dyads to raise communication and conflict resolution skills and support positive longer term relationships (Schinke et al., 2009; Velleman, 2009). Early intervention that involves both groups provides the best results (Koning et al., 2009).

New communication technologies such as KYKM, are now being used to support these programs (Newton et al., 2009a,b; Schinke et al., 2009). They are attractive to adolescents and in the case of KYKM, are out-of-school activities, therefore they provide a cost effective way to deliver alternative experiences and information to adolescents. They also provide a new opportunity to design supportive, attractive and nurturing experiences that meet the developmental needs of both groups and lead to better outcomes for participants. Research suggests that web-based programs can build social capital in terms of hope, resilience, optimism and efficacy (Luthens et al., 2008). Finally, the revolution in communication technologies has been driven by the fact that these technologies are used to meet real needs. New forms of networked technologies and the underpinning networked relationships now offer new ways for people to manage the emotional risks of negotiating boundaries, privacy and shared intimacy (De Vulpian, 2005).

Multimedia gaming resource interventions offer a portable mode of delivery that is appealing to young people. Text messaging by phone or online chat is an effective method to communicate about emotionally charged issues or to coordinate shared activities effectively (Fukkink and Hermas, 2009). Similarly, motion cartoon characters and home-made short videos (Schinke et al., 2009) have been used successfully by mothers and their daughters to amplify the emotional content and risks of situations in ways that can be accepted, discussed, thought about and learned from.

The KYKM project is a gender specific program that has been developed to meet the needs of adolescent females and their mothers and allow them to engage in shared activities. In addition, this form of delivery advocates for a consistent approach to ensuring all recipients receive the same messages. One of the advantages in the delivery of this project is that it does not rely on the school system and its overcrowded curricula or a funded community program, for its implementation. The limitations of available time of teachers, training required, and availability of facilitators and youth workers to deliver prevention programs, are often barriers for dissemination to the wider community.

## Materials and methods

Knowing you, Knowing me is an online, social media environment, built with a similar style and functionality to Facebook, but automated so that the mother and daughter interact with a virtual character, called Rose, who leads them through the intervention. In KYKM, Rose is a fairy and is presented as a “talk show” host using animated webisodes (short video episodes ranging from 2.6 to 6 min, depending on the level). The decision to use an ethereal character such as a fairy to facilitate the game capitalizes on the engagement that adolescent girls have with magical characters in television shows and cinema (e.g., Twilight, Harry Potter, Once Upon a Time, etc). As a fairy, Rose isn't a human adult authority figure and can provide advice to both the mother and daughter. This is a distinct point of difference between KYKM and other such games (Schinke et al., 2009).

**Table:** 

Key design decisions and technology development	
Content management system	The system is built around Drupal, a customizable and modular content management system (CMS). In addition to the front facing Facebook styled graphical user interface (GUI), a back end administrator system was developed to allow content experts to quickly and easily design and modify the system
Content types	Pieces of content in the system are tagged as snippets, segments of automated conversation from Rose (the fairy), messages, text messages to be automatically sent to mobile phones of mothers and daughters, media, image or video media for display on the Facebook style wall, diary questions, reflection questions for mothers and daughters for each level, daughter/mother/joint pictures, uploaded by mother and daughter and displayed alongside wall posting, levels, which organize the sequence and interactivity of each piece of content, and page, which format the individual pages of the site
Fully customizable by content experts	Knowing you, Knowing me currently comprises of 4 distinct activities: Introduction, Level 1, Level 2, and Level 3. The content of these levels have been designed to meet the needs of mothers and daughters around improving communication, relationship building, and managing risky behaviors. However, the back end administrative system has been designed to allow the removal, modification and addition of any level of content. Therefore, new levels can be added to Knowing you, Knowing me without requiring any additional software development, or a similar Knowing you, Knowing me program could be devised for Fathers and Sons (for example) using the same system. The intent is that other program developers and researchers will use this flexible system to present and evaluate their content
Presentation style and navigation interface	The front end GUI adopts a similar style and interface as Facebook wall postings, and is designed to represent the Facebook of Rose (the fairy). Mothers and daughters can post-textual responses to conversation threads on Rose's wall. They can also upload pictures of themselves, and together, which will be displayed alongside their posts, as well as other images to a gallery. A point of difference between Knowing you, Knowing me and Facebook is that Rose is an automated agent rather than a human user. As mothers and daughters leave messages on the wall, Rose then posts messages in response. Each instance of Knowing you, Knowing me has only one pair of mother and daughter as friends with Rose. However, the system is designed to run many concurrent and distinct instances of the site (as the server and network can handle)
Unlocking of content	Content experts can configure the system to unlock content as mothers and daughters progress. The current version of Knowing you, Knowing me, automatically unlocks subsequent levels 5 days after completion of the previous level. During this time mothers and daughters will receive SMSs and will have completed an agreed activity to spend time together
SMS	Rose (the system) automatically sends SMSs to the mother and daughter to remind them to practice elements presented by the program. The back end system allows any number of SMSs and day/time to send, and current program sends SMS messages at +1, +3, and +5 days after completion of the current level. These messages are different for the mother and daughter and encourage sharing of affirmations

## Results

**Figure 1:**
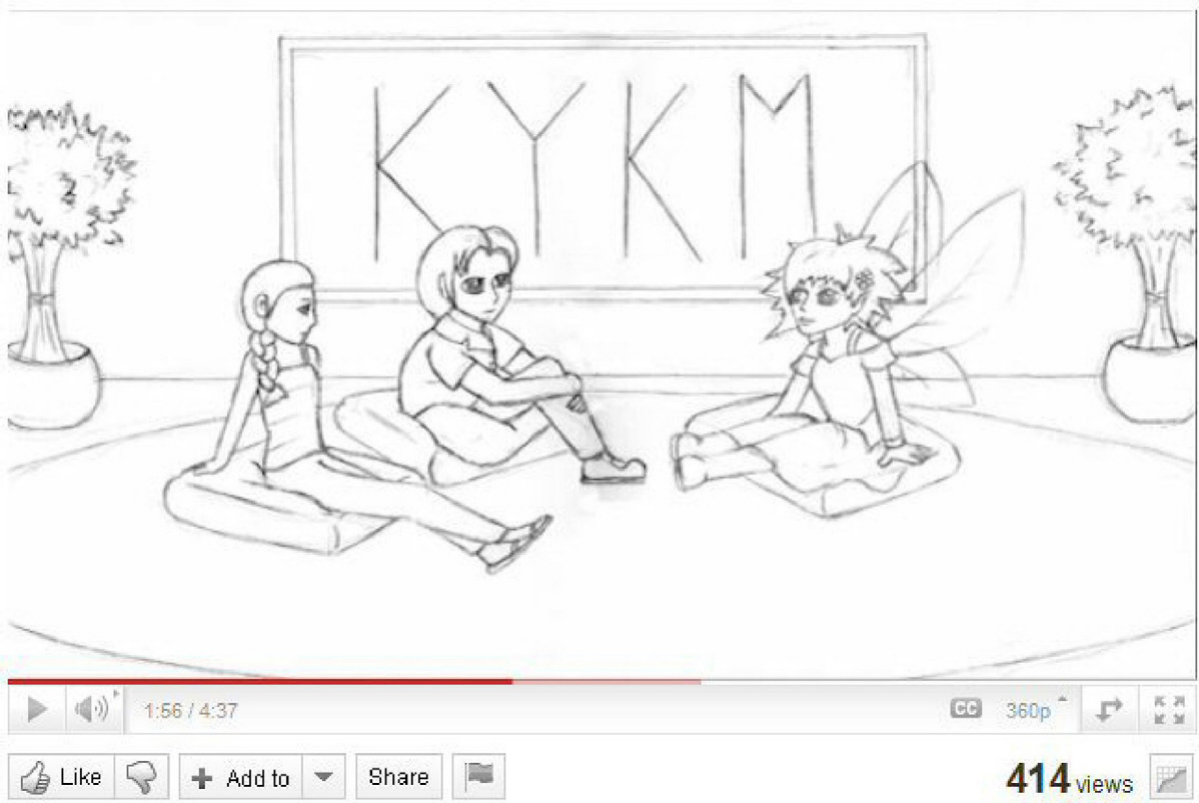
**Webisode from the Knowing you, Knowing me, talk show with Rose (right), and daughter, Lucy (left) and mother, Claire (middle)**.

From Figure 1, it can be seen that Rose is joined by a mother and her daughter, Claire and Lucy respectively, who discuss key themes of the game and provide both mother and daughter perspectives.

**Figure 2:**
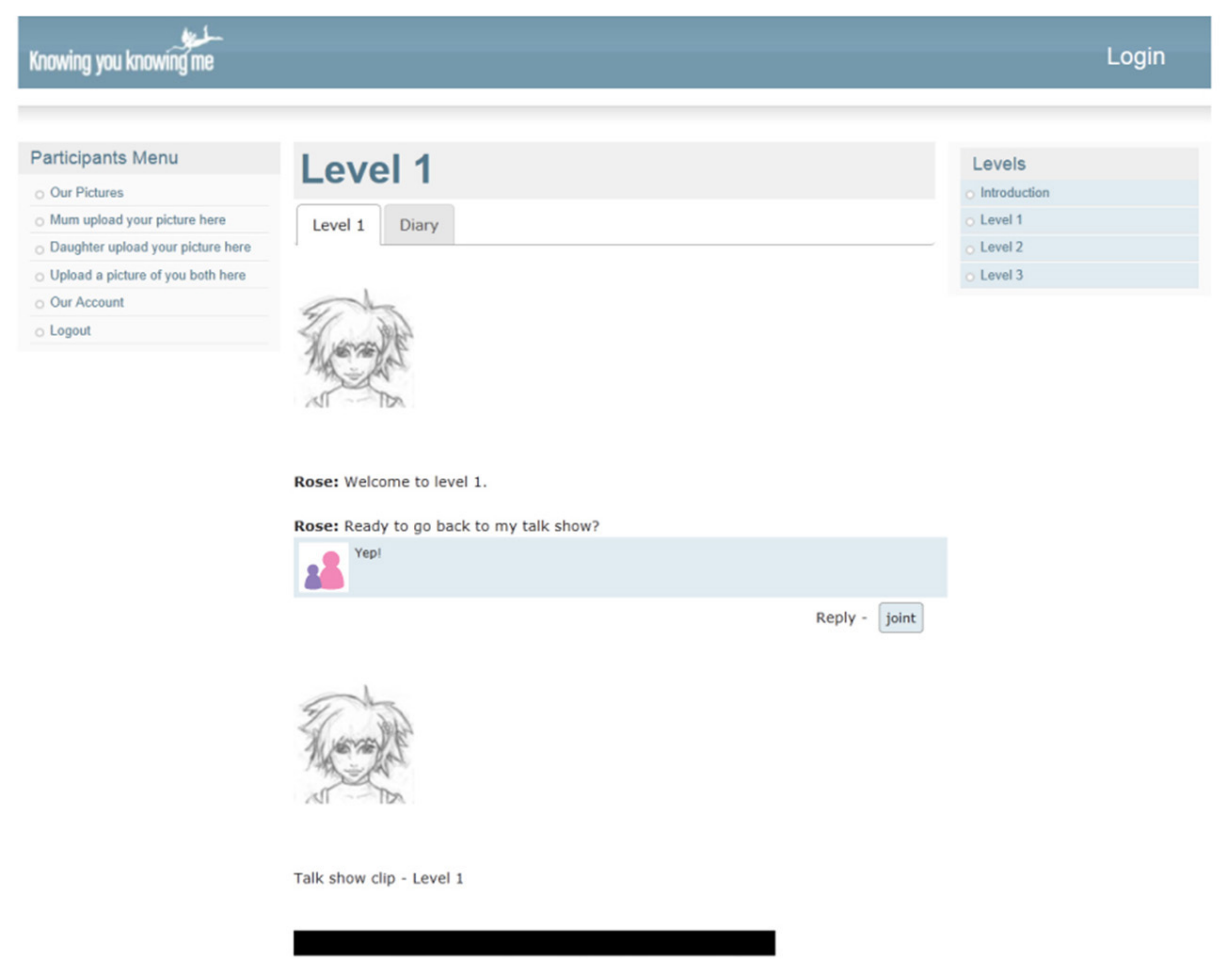
**Screenshot of Rose's Knowing you, Knowing me, social media wall, with posts from mother and daughter participants**.

Through the KYKM Facebook-styled wall, Rose asks the participating mother and daughter to post to her (respective) wall to reflect on webisodes and to also plan activities together to practice skills (see Figure 2).

Mothers and Daughters can upload images of themselves, individually and together, which are displayed alongside their posts, and can also share images in a gallery. They complete the levels together at one screen, but can also return and converse online through the program.

Participants engage in games and activities in Knowing you, Knowing me designed to integrate the educational content into their relationships. Importance is placed on the mother and the daughter listening to one another, spending time together, understanding each other's point of view, negotiating through conflict toward mutually agreeable outcomes and giving one another encouragement, support, compliments and personal favors.

The three levels (modules) in the game are:

1. Participate in effective communication,
2. Develop healthy relationships, and
3. Manage risky behavior in the social environment.

**Figure 3:**
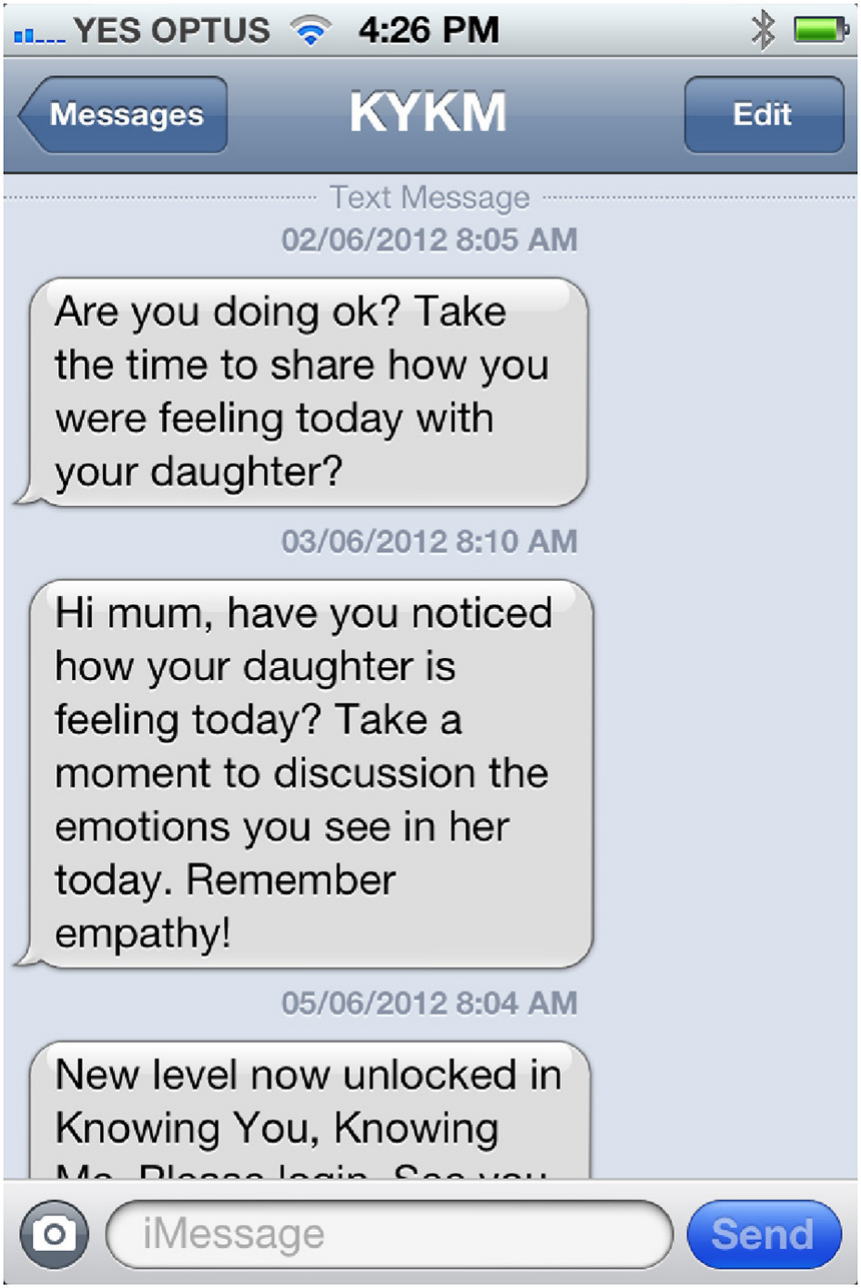
**Screenshot of mother's mobile phone showing two SMS messages and new level unlocked message**.

**Figure 4:**
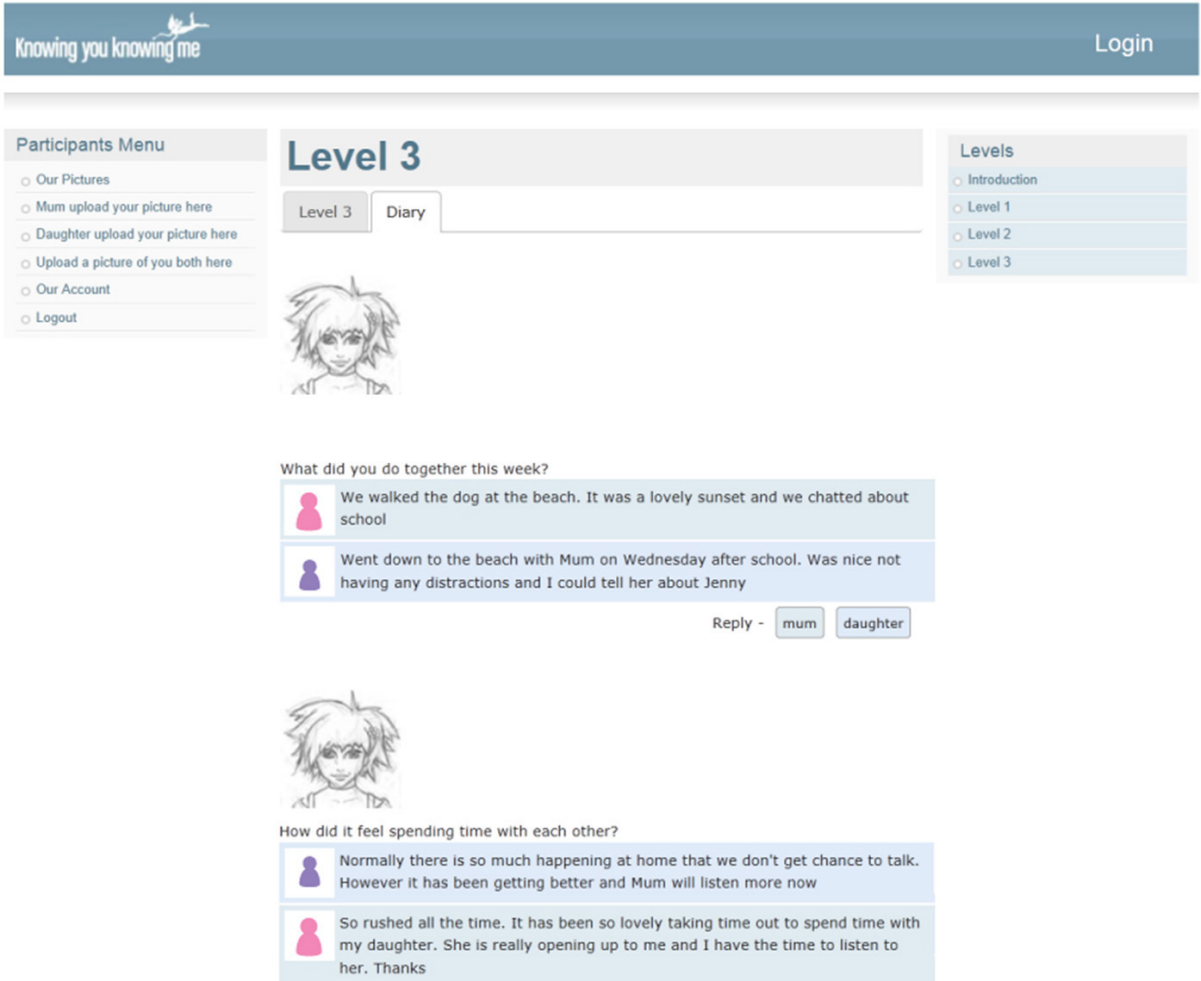
**Knowing you, Knowing me diary page with posting from mother and daughter reflecting on their experience during the week**.

**Figure 5:**
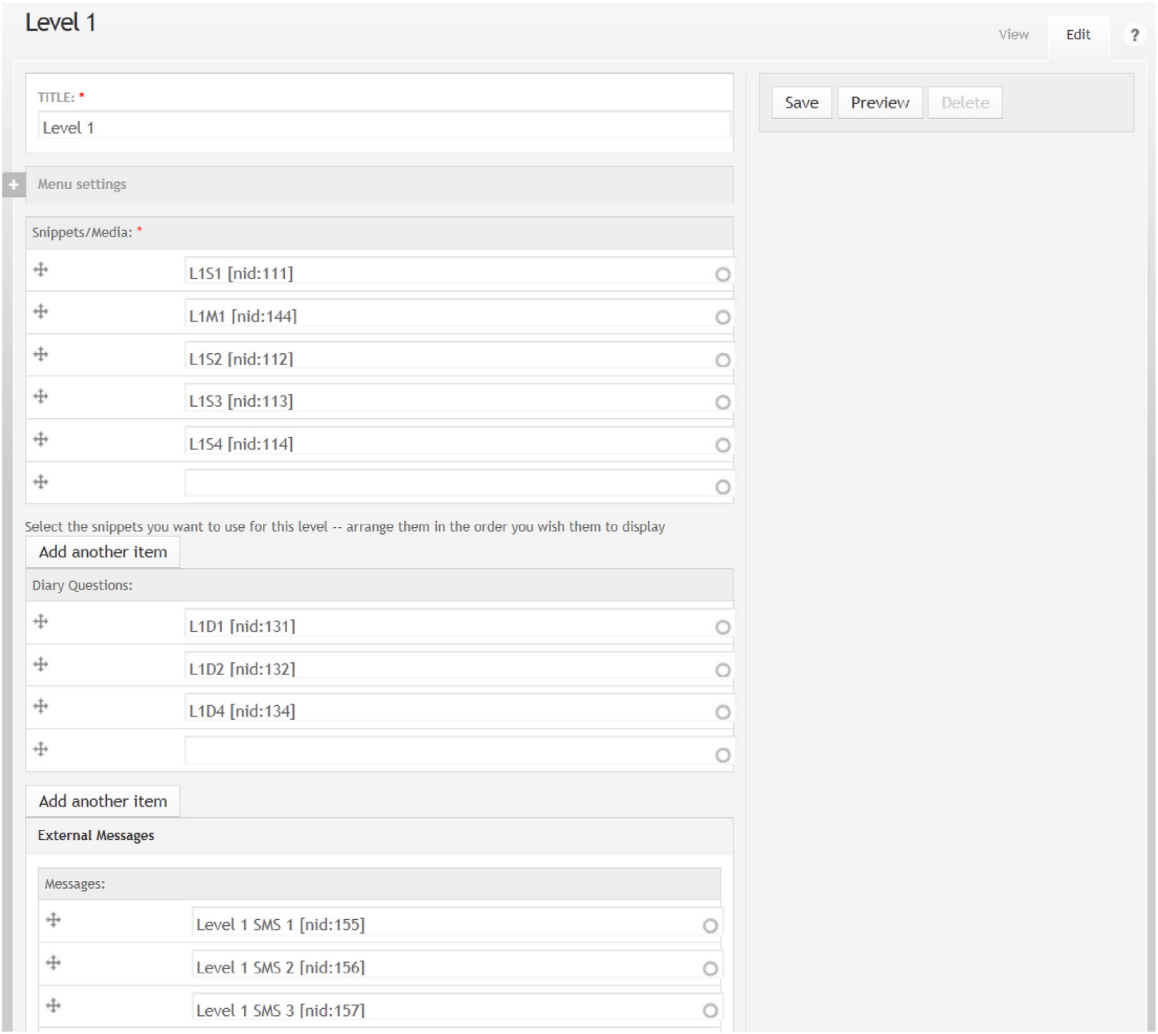
**Administrator content management system with snippets (text), media (images and videos), diary questions and SMS messages**.

The administration environment allows the research team to directly modify what, when, and how Rose discusses with the mother and daughter participants, including automatically triggering of conversations, SMS messages (see Figure 3), questions, weblinks, webisodes, diaries (see Figure 4) and unlocking of levels (see Figure 5), and new levels and content can be easily added without rebuilding the system.

## Discussion

The Knowing you, Knowing me game contains information to develop healthy interpersonal communication, respect, trust, negotiation setting limits, values, and analyzing situations. The game provides opportunities to personalize information by having mothers and daughters identify their own strengths in themselves and their relationship and identify their personal values. Additionally, there are opportunities to recognize social pressures and influences by having mothers and daughters examine images and messages used to define gender identity, mother-daughter relationships, female alcohol use and demonstrate how to be resilient to these pressures. The game provides consistent reinforcement of positive norms through the use of process praise rather than personal praise, and opportunities to learn and practice skills including refusal skills and delaying skills.

The information and activity contained in the game offers mothers and daughters a “toolbox” for handling a variety of domestic situations, from the most basic communication skills (such as listening and responding in a timely manner) to the more difficult and complex communication tasks of conflict resolution and negotiation within the scope of the problem.

A major limitation of this report is that KYKM is untested in the community of mothers and daughters that it has been developed to assist. The authors are trialing the game in Australia and would welcome collaborative approaches locally and globally. The game is available now and freely accessible to interested researchers. This report is the first step in achieving this aim and in widening the scope of the game's application.

One strength of the game design is that KYKM has the capacity to grow into many different areas beyond communication skills and alcohol use in adolescence. In a separate study using focus groups of mothers and daughters (Katsikitis et al., 2013b), valuable information was shared with the investigators with regard to future topics for Claire, Lucy and Rose to tackle (e.g., bullying, body image, friendships etc.). In addition, young adolescent boys and the relationship they have with their fathers (and/or their mothers) is also worthy of consideration. The authors recommend that this game be used as a positive psychology “intervention” in future studies so as to evaluate its properties and utility beyond the gaming circles, and investigate the possibility that KYKM may be useful in health promotion. It is anticipated that with further research in this area, KYKM has the potential to be a valid, meaningful and purposeful game into the future.

### Conflict of interest statement

The authors declare that the research was conducted in the absence of any commercial or financial relationships that could be construed as a potential conflict of interest.
